# Supplementary figures and images for: Early versus late add-on therapy in generalized myasthenia gravis: a multicenter real-world cohort study
Source: J Neurol. 2026 Feb 27;273(2):168. doi: 10.1007/s00415-026-13722-3 (PMC12948894; doi:10.1007/s00415-026-13722-3)

Early Late

A

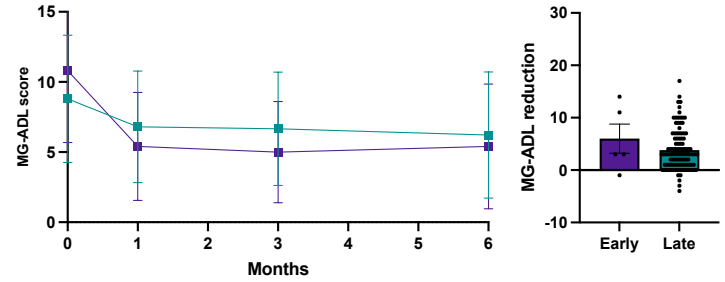

B

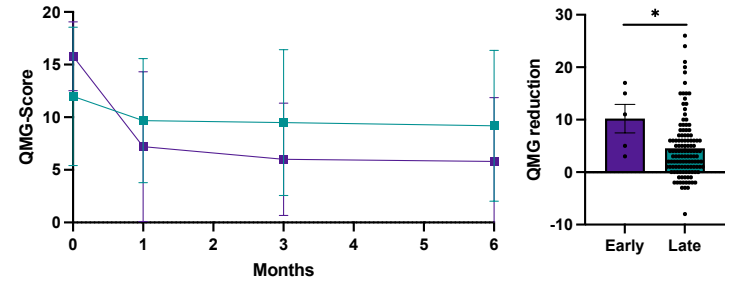

C

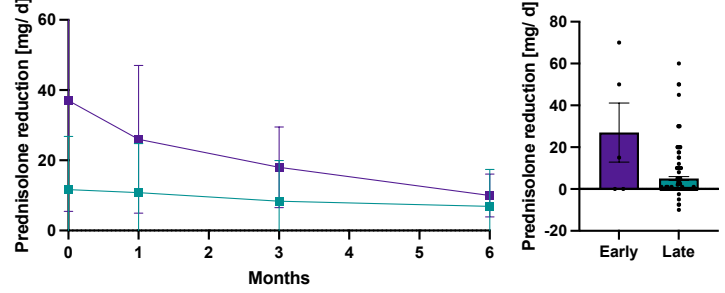

D

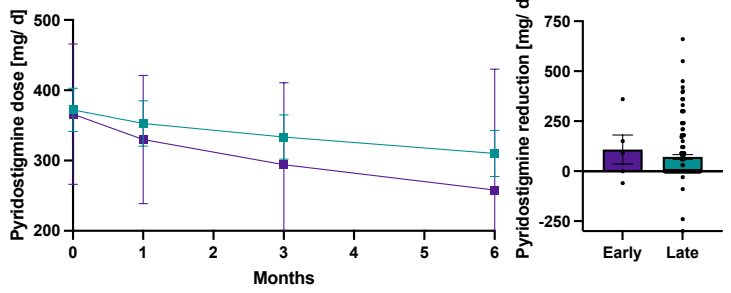

Supplemental Figure 1

Supplement: Supplementary file 1 — Supplemental Figure 1 Outcome parameters in patients undergoing treatment escalation within 13 months of diagnosis versus thereafter. This figure depicts the trajectory during the first 6 months after therapy escalation as well as the individual best response for (A) the MG-ADL, (B) QMG score, (C) daily prednisolone, and (D) daily pyridostigmine dose. Error bars represent the mean with SD. Quantitative variables were analyzed using a two-sided Student's t-test. A p-value < 0.05 was considered statistically significant. BL, baseline; MG-ADL, Myasthenia Gravis Activities of Daily Living; QMG, Quantitative Myasthenia Gravis; SD, standard deviation (PDF 476 KB) [file 415_2026_13722_MOESM1_ESM.pdf]

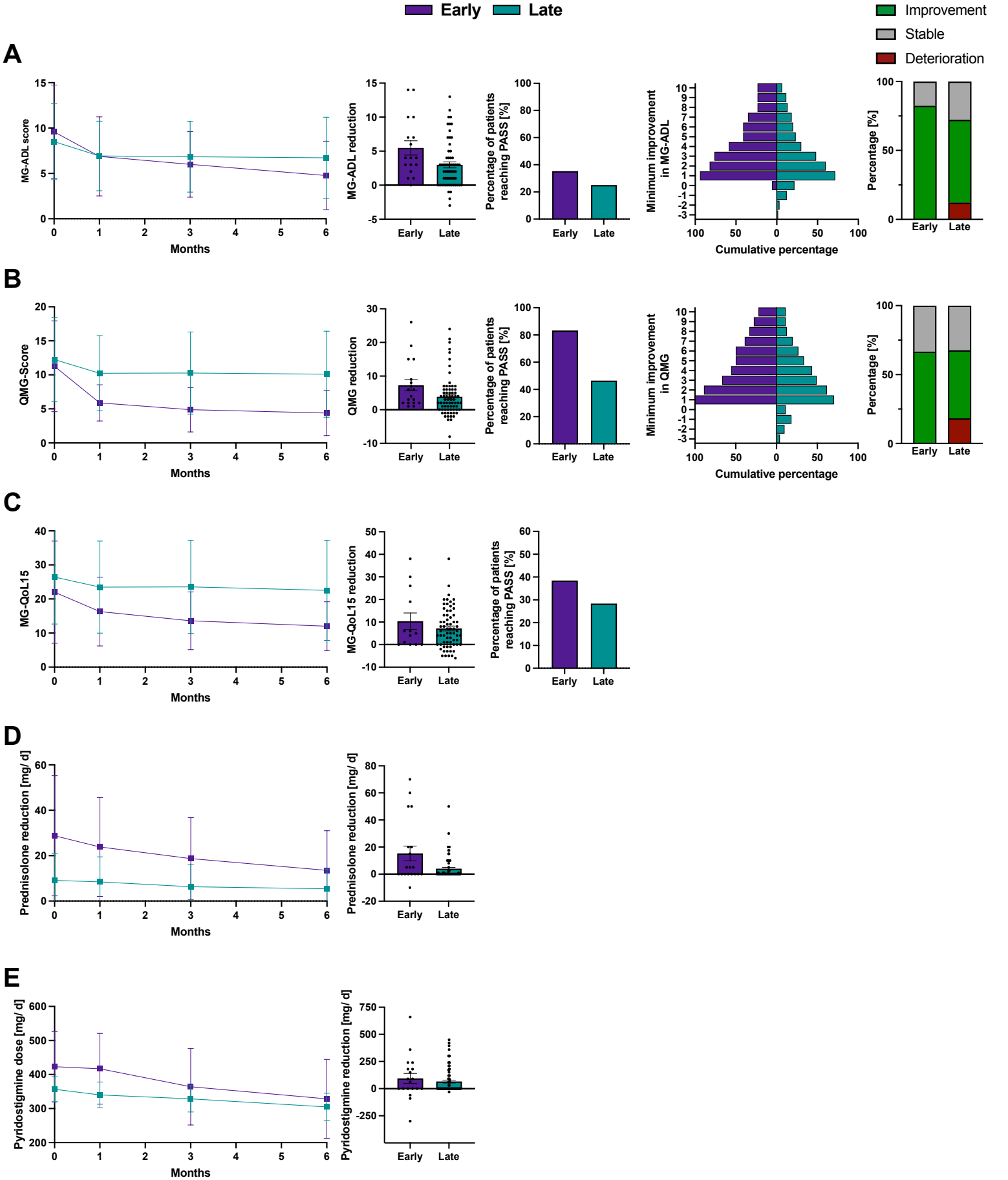

Supplemental Figure 2

Supplement: Supplementary file 2 — Supplemental Figure 2 Clinical outcomes after early (within 24 months of diagnosis) versus late (thereafter) initiation of complement C5 inhibition. The figure shows the course of key endpoints in patients treated with a C5 inhibitor, contrasting early (purple) and late (green) escalation. (A) depicts MG-ADL outcomes, showing mean scores at BL and at months 1, 3 and 6 (left), the maximal individual reduction in MG-ADL (centre-left), the proportion of patients reaching a PASS (MG-ADL score ≤ 2; centre), and the proportion achieving a clinically meaningful improvement of at least two points (centre-right). The right diagram depicts the proportion who experienced a MG-ADL deterioration (red), a stable MG-ADL (reduction of 0-1 points, grey), or a relevant improvement (MG-ADL reduction ≥ 2 points; green). (B) provides the corresponding QMG analyses, presented as mean scores within the first six months after treatment initiation, the individual maximal reduction and the proportion meeting PASS criteria (QMG ≤ 7 points). The right graph shows the proportion of patients with a QMG deterioration (red), a stable QMG (QMG reduction of 0-2 points, grey), or a significant improvement (QMG reduction ≥ 3 points; green). (C) illustrates the MG-QoL15, displaying mean scores over time (left) together with the best individual improvement and the proportion attaining PASS (score ≤ 8; right). In D, prednisolone dosing is depicted, showing mean daily prednisone doses at BL and at months 1,3, as well as 6 (left), and the greatest individual dose reduction within six months (right). (E) presents analogous data for pyridostigmine, with mean daily doses over time (left) and the maximal individual reduction achieved (right). Error bars represent mean ± SD. Groups were compared with two-sided Student’s t-tests; p < 0.05 was considered significant. BL, baseline; MG-ADL, Myasthenia Gravis Activities of Daily Living; QMG, Quantitative Myasthenia Gravis; PASS, Patient-Acceptable Symptom State; SD, [file 415_2026_13722_MOESM2_ESM.pdf]

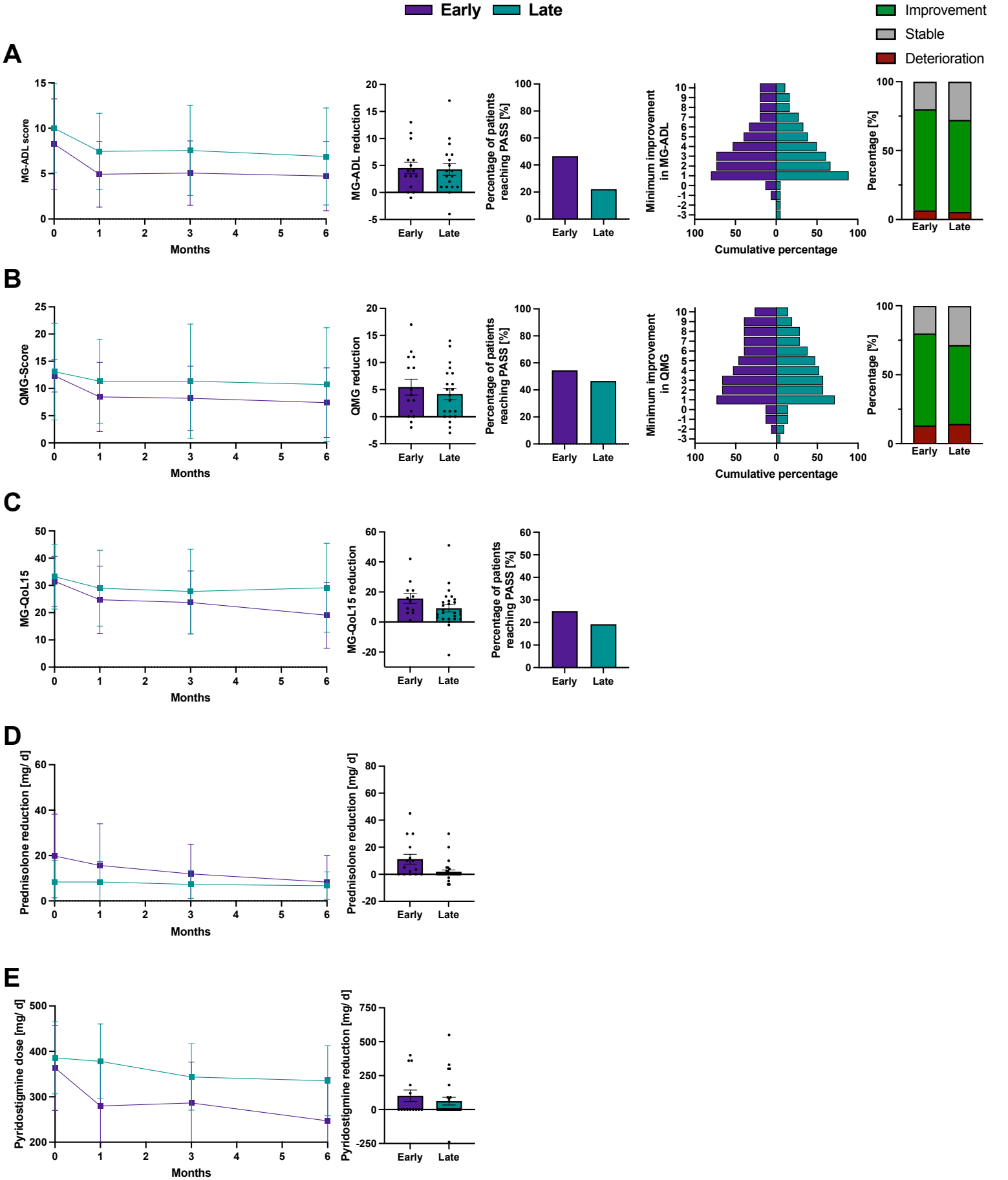

Supplemental Figure 3

Supplement: Supplementary file 3 — Supplemental Figure 3 Clinical outcomes following early (within 24 months of diagnosis) versus late (thereafter) initiation of the FcRn antagonist efgartigimod. This figure displays key clinical and pharmacological parameters in patients escalated to efgartigimod, comparing early (purple) and late (green) initiation. Panel A depicts MG-ADL dynamics, presenting mean scores at BL and at months 1, 3 and 6 (left), each patient’s maximal MG-ADL reduction (centre-left), the proportion achieving PASS (score ≤ 2; centre) and patients attaining a ≥ 2-point improvement (centre-right). The right graph shows the proportion of patients with a MG-ADL deterioration (red), a stable MG-ADL (MG-ADL reduction of 0-1 points, grey), or a significant improvement (MG-ADL reduction ≥ 2 points; green). (B) depicts the analogous QMG analyses: mean scores over the first six months, maximal individual reductions, and the proportion fulfilling PASS criteria (QMG ≤ 7). The right diagram shows the distribution of patients with QMG worsening (red), stability (QMG reduction of 0-2 points, grey), or improvement (QMG reduction ≥ 3 points; green). (C) shows mean MG-QoL15 trajectories (left) alongside the best individual improvement and proportion reaching a PASS (MG-QoL15 score ≤ 8; right). (D) reports steroid intake, displaying mean daily prednisone at BL and months 1, 3 and 6 (left) together with the maximal individual dose reduction within six months (right). In (E), pyridostigmine dosing is depicted, showing mean daily doses at BL and at months 1,3, as well as 6 (left), together with the greatest individual dose reduction within six months (right). Error bars represent mean ± SD. Groups were compared with two-sided Student’s t-tests; p < 0.05 was considered significant. BL, baseline; MG-ADL, Myasthenia Gravis Activities of Daily Living; QMG, Quantitative Myasthenia Gravis; PASS, Patient Acceptable Symptom State; SD, standard deviation (PDF 550 KB) [file 415_2026_13722_MOESM3_ESM.pdf]
